# Tour guide robot: a 5G-enabled robot museum guide

**DOI:** 10.3389/frobt.2023.1323675

**Published:** 2024-01-16

**Authors:** Stefano Rosa, Marco Randazzo, Ettore Landini, Stefano Bernagozzi, Giancarlo Sacco, Mara Piccinino, Lorenzo Natale

**Affiliations:** ^1^ Istituto Italiano di Tecnologia, Genoa, Italy; ^2^Ericsson Telecomunicazioni S.p.A., Genoa, Italy

**Keywords:** 5G, humanoids, museum guide robots, autonomous navigation, service robots

## Abstract

This paper presents and discusses the development and deployment of a tour guide robot as part of the 5 g-TOURS EU research project, aimed at developing applications enabled by 5G technology in different use cases. The objective is the development of an autonomous robotic application where intelligence is off-loaded to a remote machine via 5G network, so as to lift most of the computational load from the robot itself. The application uses components that have been widely studied in robotics, (i.e., localization, mapping, planning, interaction). However, the characteristics of the network and interactions with visitors in the wild introduce specific problems which must be taken into account. The paper discusses in detail such problems, summarizing the main results achieved both from the methodological and the experimental standpoint, and is completed by the description of the general functional architecture of the whole system, including navigation and operational services. The software implementation is also publicly available.

## 1 Introduction

In the last decades, due to technology improvements and drastically declining costs, many innovative solutions have been integrated into the cultural domain, with the aim of making art more accessible and engaging. Successful applications range from the use of mobile apps (Hanussek, 2020) to wearable devices (Alletto et al., 2015), to electroencephalograph (EEG) signals (Cruz-Garza et al., 2017) to provide personalised services such as visiting paths.

At the same time, a recent solution to engage visitors in museum tours is to use social robots. Social robots are embodied, autonomous agents that communicate and interact with humans on a social and emotional level. They represent an emerging field of research focused on maintaining the illusion of dealing with a human being.

First examples of museum tour guide robots focused on implementing autonomous navigation on mobile robotic bases (Burgard et al., 1999). Later iterations provided improved human interaction via speech (Nourbakhsh et al., 1999), head movements (Thrun et al., 1999b, Thrun et al., 1999a), display of emotions and bi-directional speech (Macaluso et al., 2005; Álvarez et al., 2010). As advances in humanoid robots increased their autonomy and affordability, they were more and more frequently employed (Shiomi et al., 2006; Falconer, 2013) for their capability of using natural gestures (SOAR, 2019).

The concept of cloud robotics (Kamei et al., 2012) introduced the idea of robots as a service, where software components such as sensing, computation, and memory are not necessarily integrated into a single system. Due to network latency, variable quality of service, and downtime, cloud robots often include some capacity for local processing for time-sensitive, low-latency services and during periods where network access is unavailable or unreliable. In particular, the requirements of a robotic system (predictable and low latency, relatively high bandwidth, reliability of connection) can benefit from the lower latency of 5G compared to 4G LTE.

The scope of the Horizon 2020 5G-TOURS project is to demonstrate the ability of 5G technology to empower different vertical use cases. One of such use cases is the development of a mobile network-enabled autonomous museum robot guide. The consistent high bandwidth and low latency of 5G networks are particularly suited for implementing robotic applications in the field with low-cost humanoid robots, that must guarantee safe and consistent operation over long periods of time. Low-cost robots require to off-load computation because of onboard processing capability and battery constraints. In this work we present and discuss the development and deployment of a tour guide robot where some demanding services are off-loaded to a remote server and to the internet via 5G. In the reminder of the paper we first summarise the hardware and software choices in its development, guided by the requirements and the characteristics of the network. We then present the operational services that are specific to the tour guide robot application in order to enable scripted explanations of the operas and interaction with visitors. We finally report the results of extensive trials in the field in two separate museums, delineating the strengths of the proposed application and what are still to be considered open issues for deployment. In particular, we discuss results on acceptability and engagement by the public, as well as autonomy and robustness.

## 2 Related work

A variety of technologies (e.g., projections, holograms, mobile apps), interactive systems (vocal interaction, touch, free actions) and accessibility (virtual tours) are nowadays available to enhance the visiting museum experience. In addition to these, over the last 20 years accessibility and attractiveness have been augmented with the employment of service robots, covering various types of applications and offering different degrees of utility and usability. Robots in museums generally fall into one of three categories: museum guides, telepresence platforms, and art installation themselves (Miller et al., 2008; Germak et al., 2015).

### 2.1 Museum guide robots

Although static robots that interact with visitors have been deployed (Bickmore et al., 2011; Pitsch et al., 2011; Yamazaki et al., 2012; Gehle et al., 2017), mobile robots are more commonly used as museum guide robots. Mobile tour guides have been first implemented as wheeled sensorized bases (Burgard et al., 1999), with the addition of communication devices such as screens, speakers, colored LEDs, arms. Subsequently, humanoids (Falconer, 2013) or more frequently semi-humanoids consisting of a mobile base and upper humanoid torso (Shiomi et al., 2006) have been employed because of their opportunity for natural interaction with humans. A summary of proposed museum guide robots, with navigation, interaction capabilities and technology readiness levels, is reported in Table 1. A more comprehensive review was also compiled in Hellou et al. (2022).

**TABLE 1:** Taxonomy of tour guide robots in terms of navigation capabilities, interaction via speech and additional modalities.

Robot name	Museum	Task	Type	Navigation	User interaction
Rhino Burgard et al. (1999)	Deutsches Museum, Bonn	Tour guide	Mobile base	Autonomous, laser + sonars	None
Sage Nourbakhsh et al. (1999)	Carnegie Museum of Natural History, Pittsburgh	Tour guide	Mobile base	Autonomous, laser + sonars	None
Minerva Thrun et al. (1999a)	Smithsonian’s National Museum of American History	Tour guide	Mobile base	Autonomous, laser + sonars	Touch display
CiceRobot Macaluso et al. (2005)	Archaeological Museum of Agrigento	Tour guide	Mobile base	Autonomous, laser + sonars	Voice, display, emotion detection
Urbano Álvarez et al. (2010)	Various exhibitions, Spain	Tour guide	Mobile base	Autonomous, laser	Voice, display, emotion detection
Robovie Shiomi et al. (2006)	Osaka Science Museum	Tour guide	Humanoid	Tracking via IR markers	Voice, gestures
Repliee Q1-Q2 Matsui et al. (2005)	World Expo 2005	Static interaction	Humanoid	None	Voice, gestures, expressions, tactile skin
Asimo Falconer (2013)	National Museum of Emerging Science and Innovation Miraikan	Static interaction	Humanoid	Semi-autonomous	Voice
Pepper SOAR (2019)	The Smithsonian	Tour guide	Humanoid	Autonomous, RGB-D + laser + sonar	Voice
Pang et al. (2018)	Chinese Heritage Centre, Nanyang	Tour guide	Humanoid	None	Voice, gestures
Del Duchetto et al. (2019)	The Collection museum, Lincoln, UK	Tour guide	Mobile base	Autonomous, laser + bumpers	Voice, display, expressions
**Tour Guide Robot**	GAM, Turin and Fondazione Palazzo Madama, Turin	Tour guide	Humanoid	Autonomous, RGB-D + laser	Voice, gestures, head movements, expressions

#### 2.1.1 Mobile bases

The earliest examples of museum guide robotic platforms include Rhino, introduced at the Deutsches Museum Bonn (Burgard et al., 1999) in 1997, and a wheeled mobile robot named Chips, introduced in 1998 at The Carnegie Museum of Natural History in Pittsburgh, which provided unidirectional narrative via speech (Nourbakhsh et al., 1999). This was followed by other iterations of the same platform, also offering limited audio-visual information (Nourbakhsh et al., 2003). The Smithsonian’s National Museum of American History introduced Minerva, the successor of Rhino, a mobile robot equipped with a moving head able to produce facial expressions to communicate emotions according to the users’s behaviour (Thrun et al., 1999a). These and later wheeled robotic platforms were able to autonomously navigate using probabilistic localization and mapping, global planning and obstacle avoidance, based on distance sensors (e.g., laser range finders, sonars, infrared). These early applications focused mostly on efficient mapping and localization in the dynamic museum environment (Trahanias et al., 2005). Network communications was first done via dedicated Ethernet (Thrun et al., 1999b), with core navigation components running onboard. In particular, in Thrun et al. (1999b), Thrun et al. (1999a) localization and obstacle avoidance would be executed mostly onboard, while people detection could be executed remotely. In our application, we show that even components that are close to the control loop of the robot, e.g., 3D obstacle avoidance, can be executed remotely via a low-latency 5G network. Later works (Trahanias et al., 2005) added Web interfaces, but communication was asynchronous in these cases, and relegated mostly to teleoperation.

Later on, the focus was moved from autonomous navigation to social interaction via speech recognition and emotional states. The CiceRobot guide robot introduced at the Archaeological Museum of Agrigento, in Italy, allowed visitors to ask questions. In order to provide coherent answers, a semantic module was developed to filter questions and retrieve useful information (Macaluso et al., 2005). Navigation was achieved with an onboard stereo camera and the placement of ad-hoc markers in the museum. The Urbano guide robot (Álvarez et al., 2010), deployed in exhibitions around Spain, was able to recognize questions and modulate speech to reflect a set of different emotional states, in addition to facial expressions. Navigation was still achieved via laser range finders, sonars and infrared. Mobile bases are still used; in Del Duchetto et al. (2019) the authors focus on high level planning and long-term operativity. Integration between the robot and the infrastructure has also recently been used; Temi (2022) is able to open automatic doors and lower the volume of other exhibitions.

#### 2.1.2 Humanoids

More recently, tour guide robots have been focusing on human-robot interaction and acquired a more humanoid appearance. Repliee Q1-Q2 used at the World Expo in 2005 had a human upper body with realistic appearance and communicated through speech, facial expressions and body language. The body was covered with tactile skin for sensing touch (Matsui et al., 2005). In a field trial at the Osaka Science Museum in 2006, four humanoid tour guides were deployed (Shiomi et al., 2006). Localization was achieved via an infrared camera tracking system and interaction with visitors via active RFID tags. Notably, Asimo was employed as a museum guide in the Japan’s National Museum of Emerging Science and Innovation in 2013 (Falconer, 2013). Recognition of the speaker among a group of visitors was explored by means of raising hands, with mixed results. Six Pepper robots were used in a pilot among three museums of the Smithsonian in Washington, answering questions from visitors and using voice and gestures to narrate (SOAR, 2019). Pepper was equipped with a combination of RGB-D camera, lasers and sonars.

Other humanoid robots include a humanoid torso, trialed with visitors in the Chinese Heritage Centre in Nanyang and offering bilingual speech interaction, people tracking via monocular face tracking and sound localization (Pang et al., 2018), and recent commercial solutions (Promobot, 2022).

### 2.2 Telepresence robots

Telepresence robots have been more widely employed, typically as a means for visitors to explore a museum or otherwise inaccessible parts of a museum by remote teleoperation. Wheeled platforms equipped with a screen and a camera are usually employed. Examples include a pilot by Csiro at the National Museum of Australia (Schulz et al., 2013), Norio (Norio, 2015) at the National Centre for Monuments in France, and multiple telepresence robots used at the Tate Museum in London for remotely exploring the exhibitions at night (Tate, 2014).

### 2.3 Experimental evaluation in the field

Most robots were tested in pilot studies in actual museums in presence of visitors during a period of days or even weeks (SOAR, 2019; Burgard et al., 1999; Nourbakhsh et al., 1999; Willeke et al., 2001; Thrun et al., 1999b), while at least one remained operative for 174 days, albeit not continuously and with different hardware and software iterations (Nourbakhsh et al., 1999) and its successors). Notably, Del Duchetto et al. (2019) traveled over 300Km for a total of 2300 tours, with an average tour duration of 4.5 minutes. The duration of most guided tours tends to be short, ranging from 2 to 10 min (Gasteiger et al., 2021). In contrast, our tour guide robot was deployed in two different venues for a period of 2 weeks each, with the first week dedicated mostly to setup and tuning, and the second week dedicated exclusively to guiding visitors through the exhibitions and collecting visitors experiences. The actual tour in both venues was active for a whole week during opening hours, and both complete tours lasted between 20 and 45 min.

## 3 Robotic platform

**FIGURE 1:**
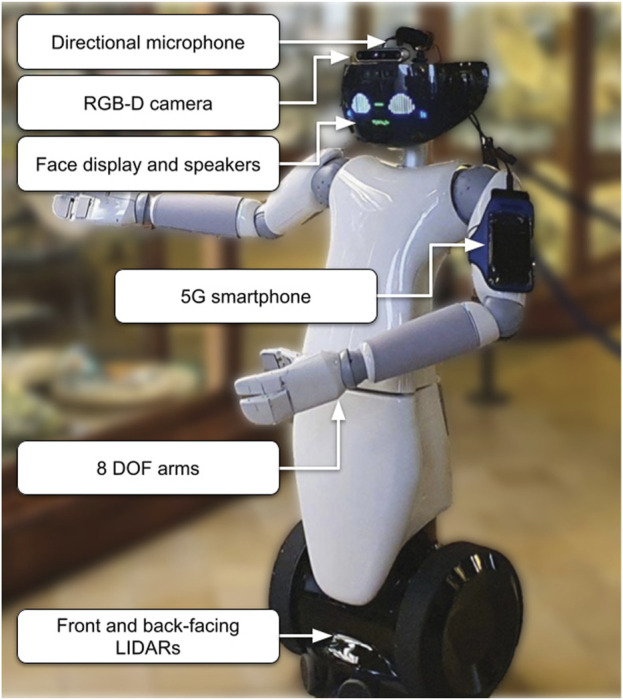
The R1 humanoid robot in the configuration used in the museum trials.

Driven by recent positive results in employing humanoids as museum tour guides (2.1.2), the R1 wheeled humanoid robot (Parmiggiani et al., 2017) was chosen as the robotic platform, due to its ability to provide both gestures and facial expressions, and smooth movement on planar surfaces. R1 (Figure 1) is an affordable mobile robot developed by Istituto Italiano di Tecnologia (IIT), which consists of a humanoid torso and head and a wheeled base. The robot has two 8 DOF arms and a 2 DOF head with a programmable RGB LED face display for displaying facial expressions, which makes it particularly suitable for interacting with visitors.

During the course of the presented work the robot sensing equipment, network hardware and onboard computing hardware went through different revisions, before reaching the final configuration used in the experimental trials. Originally, the robot was equipped with two RPLIDAR A2 laser range finders mounted on the base, between the wheels, facing forward and backwards; one Realsense D435 RGB-D camera was mounted inside the face, and two embedded PCs (a NUC inside the robot’s base and a Nvidia Jetson Xavier AGX mounted inside its torso), as well as a custom FPGA inside the head for controlling the LED display. A Mikrotik hAP ac router located in the torso connects all decentralized robot components and allows remote communication, via a 5G-enabled smartphone connected via USB, which was fixed on one arm with an armband. Some devices were not used, such as a stereo camera system located in the eyes of the robot.

During the course of the project, the two laser range finders were upgraded to RPLIDAR A3s with a maximum usable range of 25m; the RGB-D camera was upgraded to a Realsense D455, which offers a larger field of view as well as increased depth accuracy. The larger field of view, in combination with head movements, makes it possible to enhance visual awareness of dynamic obstacles close the robot for added safety, while also allowing to plan smoother trajectories around them.

## 4 System architecture

**FIGURE 2:**
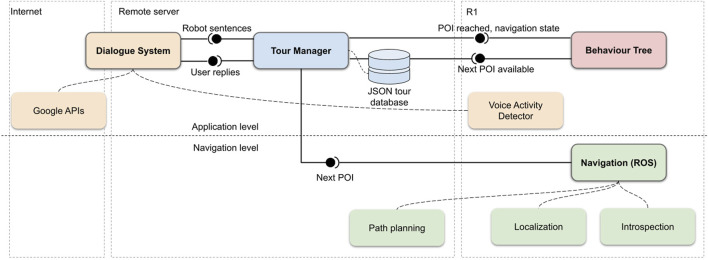
High-level overview of the application’s main components and their interfaces. The components are organized by abstraction level and by physical placement. The bottom row represents the navigation level, while the top row contains the application-level components. The main component, Tour Manager, is executed on a remote machine, as well as the Dialogue System, that communicates with Goole APIs on the internet. The Behaviour Tree is run locally on the robot, while the Navigation component is split between the robot and the remote machine. Components in green constitute the navigation system, components in yellow are part of the dialogue system.

This Section describes the components of the actual tour guide robot application. A graphical overview of the application is reported in Figure 2. It shows the components residing onboard the robot, externally on a local server and remotely over the internet.

From the software standpoint, the R1 humanoid robot is based on a middleware developed by IIT called YARP (Metta et al., 2006). All navigation and operational services are built on top of the YARP libraries, which are responsible for managing the data exchange across the network. YARP implements communication through special objects, called ports, which deliver messages to any number of observers (other ports). The computation can thus happen locally, i.e., on a single machine, or can be distributed across any number of machines, each of them running multiple processes, using any of several underlying communication protocols. The YARP middleware implements a plug-in system that allows to add data compression methods to connections, and an efficient method for inter-process communication through shared memory. These features were fundamental to optimize the data flow within the system.

For autonomous navigation, on the other hand, we used components from the ROS 1 eco-system (ROS, 2023). Communication within ROS components residing on the same machine was done through ROS topics, while communication between YARP and ROS components and between ROS components residing on different machines exploited existing YARP-ROS interfaces (Randazzo et al., 2018). In particular, ROS Noetic was used in the proposed application.

The software implementation for the tour guide robot application is publicly available (guide robot source code, T 2023).

In the remainder if this Section, we will first describe the implementation of the autonomous navigation services (localization, map creation, path planning) necessary for the movement of the robot, then the operational services that constitute the actual tour guide application.

### 4.1 Network configuration

**FIGURE 3:**
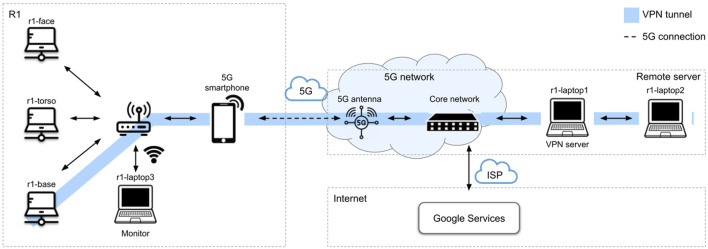
Network configuration for the museum trials. Onboard the robot, all machines are connected to a router. On the remote side, machines are also connected via a router, which is in turn connected to the internet. The robot is connected to a commercial 5G network via a tethered smartphone; the remote side is connected to 5G via an infrastructure of deployed antennas. A VPN tunnel is created between the PC in the robot’s base and a remote machine acting as VPN server.

The network configuration is shown in Figure 3. Onboard the robot, the three machines (r1-base, r1-torso, r1-face) are connected through an internal router. During experimental tests, an external machine (r1-laptop3 in Figure 3) was also connected via Wi-Fi directly to the router, and was used to monitor operation, as well as launch and restart parts of the application.

A series of compromises were made in order to allow R1 to connect to the 5G infrastructure. Initially, the use of an integrated router-5G modem was explored, but no commercially available solution was compatible with the application requirements. For this reason, a compromise solution was implemented with the addition of a 5G-enabled smartphone connected via USB to the internal router in tethering mode. The smartphone was fixed to one of the arms with a sport armband.

As with most robotic frameworks that support distributed communication, YARP requires all the hosts to belong to the same subnet and have a reachable IP address. While this is normally achieved in a laboratory setting, it is not the case on a commercial mobile network. To overcome this difficulty, a VPN tunnel was initially set up between the robot’s internal router and the remote host, but the Mikrotik router, as well as similar alternatives, restricts the maximum bandwidth for VPN traffic to 100Mbps. For this reason, the VPN tunnel was moved in between the PC in the robot’s base and the remote host (r1-laptop1, as outlined in Figure 3). Computationally-heavy services (crowd detection, gaze detection, obstacle avoidance) were off-loaded and distributed over the remote machines (r1-laptop1, r1-laptop2 in Figure 3).

### 4.2 Navigation services supporting robot operation

For autonomous navigation of the robot, traditional laser-based methods for indoor planar environments were chosen. These are available as off-the-shelf components in most robotic frameworks. While initial studies were devoted to assess the need for learning-based methods for visual navigation, preliminary experimental tests showed how off-the-shelf methods were robust enough in practice to guarantee safe navigation, due to the relatively static and structured nature of the museum environment.

Autonomous navigation services are executed both onboard and externally to the robot, in order to take full advantage of remote computational power and to study the feasibility of offloading a core capability such as autonomous navigation over a 5G network. In particular, localization (Section 4.2.1) is executed onboard the robot. This is motivated by the fact that localization is a vital component of navigation and requires as little delay as possible for safety, while at the same time being relatively computationally light. Path planning 4.2.2 is also a service that needs to be responsive. However, for safely purposes we implemented 3D obstacle avoidance which requires ray-casting (see 4.2.2). With our setup, the path planning pipeline was not able to run on the onboard embedded PCs without missing computation steps, because of the heavy computational load. For this reason, the whole path planning service was moved to a remote machine, posing the issues of network delay and bandwidth. The bandwidth issue was addressed through sensor data compression (Section 4.2.4) and safe operation was possible due to the low latency of the 5G network.

#### 4.2.1 Localization and mapping

SLAM for map creation, we used the off-the-shelf gmapping SLAM package provided by ROS, tuned for the R1 platform. This was possible due to the fact that museums are typically static environments and that the mapping process could be done beforehand, when the museum is empty, before or after opening time. Due to the large scale of the map, the relative lack of geometric features (the walls are mostly bare) and lack of intermediate loop closings in one of the two venues, we found that a low-cost laser range finder with 5 m of usable range was not enough to obtain an accurate map without the help of temporary obstacles to be put in the environment during mapping (e.g., small boxes in our experiments) to act as additional features. However, the second iteration of the robot, equipped with LIDARs with a usable range of 18 m, was able to map both museum floors without any additional infrastructure.

Localization for localization inside the map we also used ROS, in particular the AMCL package which implements 2D LIDAR global localization, that was also tuned to the sensors of R1. AMCL proved to be robust to high amounts of occlusion caused by visitors surrounding the robot. This was tested in simulation by embedding a crowd simulator inside the system, and measuring the localization performance with varying amounts of people cluttering the laser range readings.

**FIGURE 4:**
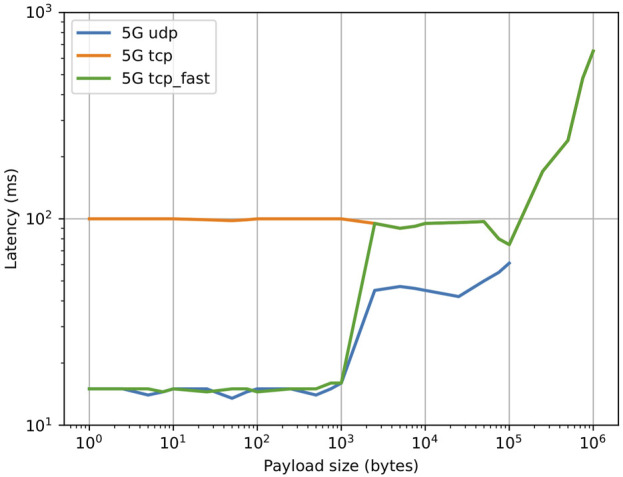
Effect of payload size on application level latency for two machines connected via 5G and through a VPN tunnel with a default configuration, using different YARP transport layers.

Localization introspection on the other hand, particle filter localization is known to not be robust to highly symmetric environments (Abrate et al., 2013), in particular in the case of robot kidnapping or re-localization. In order to detect such cases when the robot pose estimate becomes inconsistent, an introspection module was implemented as shown in Figure 4 that compares different streams of localization both in space and time to detect inconsistent pose estimates. The implementation is based on the one proposed in Antonante et al. (2021). Two localization streams were investigated for the trials: laser-based AMCL and open-loop wheel odometry. For each localization stream, independent consistency checks are done separately on position and orientation estimates. These tests are executed both on the same stream through time (e.g., by comparing estimates at successive timesteps and detecting large temporal discrepancies according to the robot motion model), and across different streams (e.g., comparing the output of laser-based localization and open-loop odometry displacement at the same timestep). Independent fault counters are updated according to found inconsistencies, and are then summed in order to predict a localization state: consistent if there are no faults, warning state if there are less than a given threshold number of faults, error state if there are more that the threshold number. If the output of the introspection module is an error state, navigation is stopped for safety and the robot requests assistance from the operator. This is handled by the behaviour tree component (4.3.2). During experimental tests in the lab, a third visual localization stream was used. However, during the final trials, it was found that LIDAR-based localization was robust enough in practice to not require the use of the ORB-SLAM2 stream.

#### 4.2.2 Robust planning and obstacle avoidance

Path planning and obstacle avoidance services were implemented using off-the-shelf algorithms from the ROS move_base package. In ROS, motion planning of the robot mobile base is implemented as a dual layer architecture consisting of a global and a local planner. The global planner plans an optimal trajectory to a given goal according the obstacles in the map and the local planner follows the trajectory while avoiding dynamic obstacles acquired from the robot’s distance sensors, generating velocity commands for the base. move_base keeps track of fixed and dynamic obstacles via occupancy grid-maps, called *costmaps*. These are implemented as a series of composable layers. A* was used as a global planner and the Dynamic Window Approach (DWA) was used for obstacle avoidance.

Since the robot is moving around and close to people, it is necessary to detect dynamic obstacles in the full 3D space around it. The voxel layer was used in order to generate a 3D voxel map around the robot and extending up from the ground plane to the robot’s height; the voxel map is then projected onto the ground plane, and if more than a set number of voxels (2 in our experiments) are projected down onto the same map cell, that cell is marked as an obstacle.

A YARP node was implemented that, given the planned trajectory for the robot, moves the head so as to look ahead at the next point on the trajectory that lies on a circle of a given radius around the robot frame. This helps mitigate the narrow field of view of the camera when the robot is turning and more in general helps detecting dynamic 3D obstacles that are above the laser range finder and on the robot’s path during movement (Nobile et al., 2021).

As an additional safety layer, certain areas of the map (e.g., areas that are too close to the operas) are masked out and result as non-feasible during planning. This was achieved using the costmap_prohibition_layer. The prohibited areas were marked manually after map creation, based on the layout of the tour.

Another important aspect of navigation that is needed to make the robot’s movement more natural is the ability to recover from situations where the planning has failed. We used the recovery behaviours defined by move_base. After planning failed for three consecutive times due to obstacles still present around the robot, a recovery behaviour clears all remaining dynamic obstacles from the costmap. Although this could be potentially dangerous if the robot is in fact close to an obstacle that is not seen by its sensors, the combination of head scanning and local planner made the navigation safe enough in practice.

#### 4.2.3 Crowd detection and gaze detection

A YARP OpenPose (Cao et al., 2019) node was run remotely for the purpose of checking that the next navigation goal (the next opera in the tour or the starting/docking pose) is not being occupied by one or more visitors. This is checked at the application level by the behaviour tree component (see Section 4.3.2).

Additionally, in order to increase the robustness of speech recognition in the wild, a gaze estimation YARP node (Lombardi et al., 2022) was also employed that detects the presence of visitors looking directly at the robot’s face (camera). The gaze detector takes as inputs the presence of people in front of the camera from OpenPose, as well as a subset of the face keypoints representing each person’s face, and detects eye contact from people actively looking at the camera via a *Support Vector Machine* (SVM) classifier. The combination of detected speech by the voice activity detector (Section 4.3.3) and detected gaze (indicating attention) are used to robustly detect a visitor asking a question. In addition, the robot’s head actively tracks the closest visitor facing the robot, to provide more natural interaction during speech.

#### 4.2.4 Sensor data compression and choice of carrier

When designing a networked robotic system, transmitted data could be broadly divided into command and sensor data. Command data (e.g., velocity commands) are typically sent from an external controller to the robot and can be both sporadic or repeated at a constant rate. Sporadic commands or *Remote Procedure Calls* (RPCs) are used to set the value of a specific control parameter and require an acknowledge/status reply, since their loss could cause a catastrophic failure of the system. For this reason, sporadic commands in YARP typically use the YARP TCP carrier. to an external controller On the other hand, in the case of repeated commands only the most recent one is important. Missing a command may cause a glitch, but the system will recover when the next command is received. Streaming commands typically employ the YARP TCP_fast carrier. Command data are typically small in size (below 50 bytes on average) and are required to have low latency.

Sensor data, when transmitted over the network, are usually sent by the robot at a constant rate and can be large in size. Only the last received value is usually meaningful: similarly to streaming commands, data packets can be sporadically lost in the queue, without causing a catastrophic failure of the system. However, prolonged packet loss (e.g., over a 100 ms time) will cause issues. In YARP, the transmission of sensor data typically relies on the UDP carrier, with the only exception of compressed image data which may use different protocols, depending on the specific compression algorithm. The YARP MJPEG carrier, for example, is based on get HTTP requests and uses TCP at a lower level. Different sensors generate different types of traffic: the size of the array of encoders measurements ranges between 0.8 and 2KB, a LIDAR scan between 1 and 20KB, a compressed camera frame between 50 and 500 KB. That these values are substantially larger than the ones corresponding to command data.

After the installation of the 5G coverage in the areas inside both museums that were designated to be the site for the trials, preliminary functional tests were carried out in one of the two sites to evaluate the viability of 5G connectivity in comparisong with pre-existing connectivity. The tests evaluated the performance of the system in terms of video quality, smoothness of movements, end-to-end delay of audio and video streams (Table 2). The tests were carried out using the project’s dedicated 5G connectivity, existing 4G connectivity, and (where available) an infrastructural Wi-Fi connection. The results showed a marked improvement in both qualitative and quantitative performance using mobile networks compared to Wi-Fi. Using Wi-Fi connectivity, the RTT showed a large standard deviation of 3.5s. The bitrate under these conditions was also significantly lower than either 4G or 5G at about 0.54Mbps uplink. On the other hand, using mobile connectivity, the variance in RTT was much more contained, with an average 0.125s for the 4G network, and between 60 ms and 0.088s for the 5G network. However, 5G showed a lower standard deviation in RTT. Uplink with 5G showed the best results at 2.2Mbps of average bitrate; the variance was similar to 4G and Wi-Fi. The difference between 4G and 5G was also evident above all in a qualitative evaluation of the stability of autonomous navigation throughout the areas where the tests were carried out.

**TABLE 2:** Preliminary evaluation of data transmission performance to/from the robot in the museum environment. For 5G and 4G, the robot is connected to the infrastructure via the tethered smartphone. We report mean and standard deviation values (standard deviation in parentheses).

	Wi-Fi	4G	5G
Uplink (Mb/s)	0.54 (1.31)	1.75 (0.76)	2.20 (1.25)
Downlink (Mb/s)	0.55 (1.85)	6.11 (1.50)	6.10 (2.25)
RTT (s)	0.15 (3.5)	0.125 (0.085)	0.088 (0.032)

**FIGURE 5:**
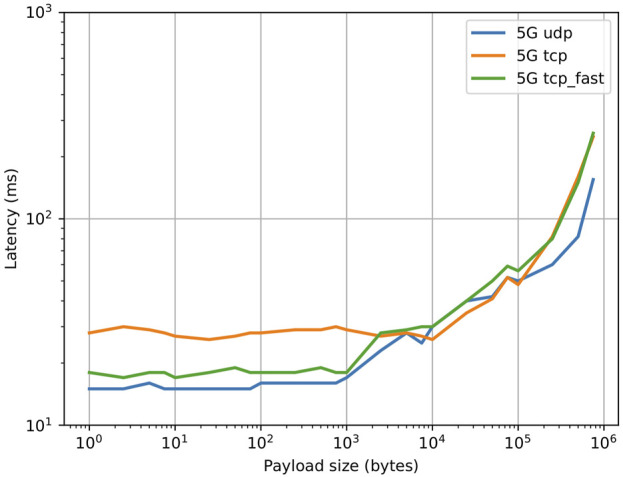
Effect of payload size on application level latency for two machines connected via 5G and through a VPN tunnel, after VPN optimization.

**FIGURE 6:**
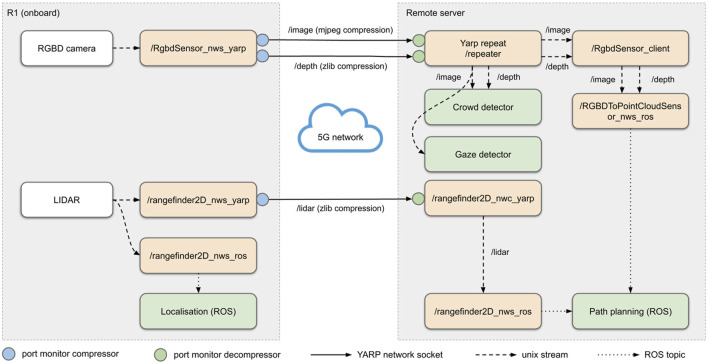
Management of sensor streams over the network via YARP repeaters and data compression via port monitors. Yellow blocks represent YARP network wrapped device drivers; white blocks represent sensors; green blocks represent macro components (either ROS-based or YARP-based). On the remote side, YARP ports are multiplexed to different subscribers via a YARP reperater, to avoid data duplication.

Preliminary tests were conducted to characterize the performance of the different YARP carriers in the application scenario, where two remote machines (one located onboard the robot and one offboard) are connected via 5G and are connected by a VPN tunnel (OpenVPN). The results are shown in Figure 5 and show the effects of adding a VPN tunnel. Up to a payload size of ∼ 1KB, UDP and TCP_fast exhibit a similar latency of 15 ms (comparable to the base network latency in the test), while TCP exhibits an higher latency of 100 ms. When the payload size increases above 1KB, the latency for TCP_fast jumps to close to 100 ms, while the latency for UDP keeps increasing up to 100KB, at which point YARP is unable to reorder fragmented packets due to internal buffer limits. It was experimentally found that by careful configuration of the VPN it is possible to reduce TCP latency. In particular, the TCP_NODELAY flag was enabled, which disables the internal Nagle’s algorithm by sending data as soon as possible instead of accumulating it until a TCP ack from previous packets is received. Additionally, the values for MTU and MSS were optimized and the preferred VPN transport protocol was set to UDP. The results are shown in Figure 6. It is possible to see that the latency for both YARP TCP carriers was greatly reduced to values close to the UDP carrier.

**FIGURE 7:**
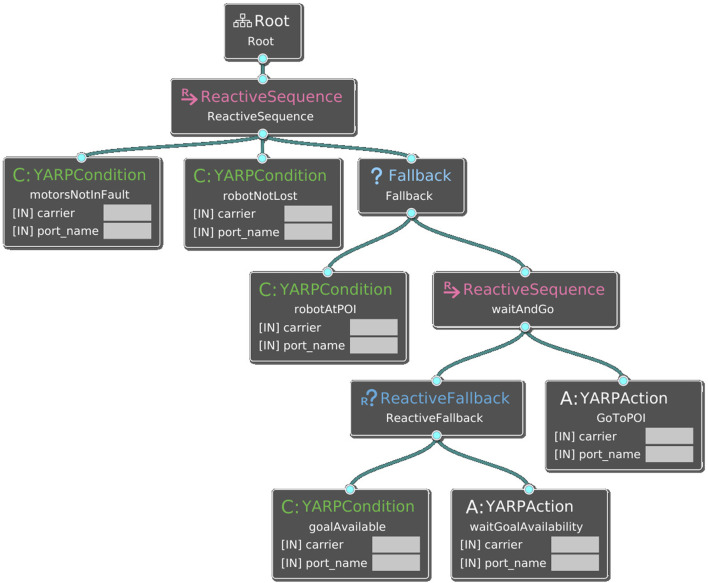
The behaviour tree for the tour guide robot application. Conditions are shown as green leaves; actions as white leaves. Compositional nodes are shown in blue (fallback) and purple (sequence).

The main challenge in developing a networked robotic application over a 5G mobile network based on the *Non-Stand Alone* (NSA) architecture is the asymmetric bandwidth, where the download bandwidth is significantly larger than upload. On the contrary, a robotic application has the opposite requirement of uploading large amounts of sensor data from the robot, making the uplink an upper bound for the amount of transmitted data. Three solutions were adopted: careful throttling of sensor data, data compression, and routing through YARP repeaters and a network wrapper architecture. Figure 7 shows the network data streams in our tour guide robot.

Throttling: both LIDAR and RGB-D camera streams were throttled to 10 Hz. This was possible due to the fact that RGB-D images are only used for obstacle avoidance by the ROS navigation system and for higher level tasks that do not require a higher frame rate (crowd detection, gaze detection).

Compression: all networked sensor data is compressed via YARP port monitors. Port monitors are extensions that can be plugged into the port of an existing YARP module, and are loaded as shared libraries (or run-time scripts); they can modify outgoing or incoming messages directly via shared memory access, thus introducing no communication overhead. In particular, RGB image data is compressed with a lossy MJPEG encoding, while distance sensor data (depth images and LIDAR) are compressed with lossless zlib compression. Compression and decompression are executed by port monitors on the output and input ports of each sensor stream respectively (Figure 7).

Repeaters: to avoid data duplication over the network due to multiple clients connecting to the same sensor data, a YARP repeat node intercepts RGB and depth images and relays them to the different clients on the same machine via Unix streams.

With the combination of three strategies the bandwidth occupied by sensor data was reduced to less than 50Mbps, well below the maximum upload bandwidth of the system. Network Wrapper architecture: YARP Network Wrapper Servers (NWS) and Clients (NWC) extend device drivers functionalities to remote devices. A NWC is a device driver that implements the same interfaces of a normal device, but instead of being connected to a physical device, it is connected to a NWS, or equivalent for different middlewares such as ROS. A NWS is a thin wrapper that forwards the interfaces of a device driver to a client. This makes possible to access devices in a seamless way, whether they are located locally or remotely.

### 4.3 Operational services

The components which constitute the application layer of the tour guide robot are the tour manager, the behavior tree and the dialogue system. The tour manager and the dialogue system run externally to the robot and communicate with it through the 5G network. On the contrary, the behavior tree is computationally lightweight and is executed onboard, in order to be able to maintain control of the system in case of network connectivity issues.

#### 4.3.1 Tour manager

The tour manager component is in charge of orchestrating the tours. A tour is organised as a map of the venue and a collection of *Points Of Interest* (POIs); each POI represents a 2D pose in the map, and a series of actions to be executed when the robot reaches each POI. These are stored in a configuration file in the JSON format. A special POI is the starting/ending point for the tour, in which users can select the tour language and also acts as a docking location for battery recharging.

At each POI, the robot will stop and execute the actions specified, which can be of three kinds:

Speak: the robot explains the opera or asks the visitors for input. The text is specified in the configuration file (the JSON tour database in Figure 2) and is sent to the dialog manager to be converted to speech.

Dance: this action can be any movement of the robot’s joints (e.g., moving the arm to a particular pose in order to point at an opera during an explanation; turning the head to point at an opera). Dance movements can be executed in series or in parallel with other actions such as speaking.

Signal: this is a special action which is used to switch language when a new language is selected at the start of the tour or to introduce a pause in the tour.

The tour manager component is called by the behaviour tree (Section 4.3.2) whenever the robot has to move to a new Point Of Interest (POI) and no hardware or localization errors are triggered (GoToPOI action within the Behavior Tree 9). The tour manager fetches the next POI from the tour configuration, communicates it to the ROS navigation component, monitors the state of navigation when asked by the behaviour tree and, when a new POI is successfully reached, it orchestrates the actions to be executed at that particular POI (speak, dance or signal actions) as described in the tour configuration.

#### 4.3.2 Behaviour tree

The behavior tree interacts with the navigation and the tour manager components (Section 2). It manages navigation faults, both hardware and software, by checking for fault conditions and calling suitable actions. In addition, it triggers the action of moving to the next POI in the tour manager. *Behaviour Trees* (BTs) are a graphical model for reactive, fault-tolerant task execution (Colledanchise and Natale, 2021). A BT is modeled as a directed rooted tree, with the internal nodes representing behavior compositions and the leaf nodes representing actuation or sensing. Execution starts from the root node, that sends activation signals to its children. When activated, children nodes are executed left to right in sequence or in parallel. A child node returns to its parent a status that can be either *Success*, *Failure* on completion, or *Running* if it is still executing. The tree is organized hierarchically, with its leaves representing either conditions (e.g., triggers for specific behaviors) or actions.

**FIGURE 8:**
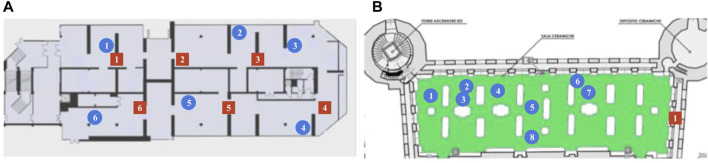
Location of the points of interest (blue circles) and DOT antennas (red squares) for the GAM museum **(A)** and Palazzo Madama **(B)**. Environment dimensions are ∼60×20m for the GAM museum and ∼30×12m for Palazzo Madama. Note that the CAD maps are not necessarily accurate to the actual dimensions and layout of the actual environment.

The behaviour tree for the tour guide robot is shown in Figure 8. The main functions of the behavior tree component are sending the next point of interest to the navigation component (this is done by triggering the “GoToPOI” action) and the reactive management of hardware and software failure cases. For instance, if during the navigation the robot is lost (”Condition: robotNotLost”, Figure 8), the behaviour tree stops navigation and the robot issues a warning via speech. Fault conditions implemented by the application are: running internal diagnostics that monitor the status of the motors to detect overcurrent faults, calling the localization introspection module for checking localization inconsistencies, calling the crowd detector for checking availability of the POI. Other reactive behaviors that were implemented but not used during the final trials were: checking the state of network connectivity, checking battery charge, checking whether the robot is being touched by a user.

#### 4.3.3 Dialogue system

The dialogue system handles all speech interaction and is composed of three blocks: the speech recognition system, the dialogue manager, and the speech synthesizer. The speech recognition system is activated by specific actions executed by the tour manager (e.g., the robot asks the visitors if they have questions about the current artwork).

Speech recognition system: a *Voice Activity Detector* (VAD) based on libfvad (Libvfad, 2023) runs locally on the robot and is used to cut snippets of live audio containing candidate sentences (questions and answers by the visitors) from the continuous audio stream captured by the microphone. The extracted audio is then transmitted to Google Cloud Services over the internet. The reply is a textual transcription of the sentence, which is then analyzed by the second block of the processing pipeline, the dialogue manager.

Dialogue manager: The dialogue manager is also based on a cloud application provided by Google, Dialogflow. Dialogflow behaviour is similar to a finite state machine, whose transitions between the different states are triggered by intents, i.e., the sentences received by the transcribed text received from the speech recognition system. The rationale for the choice of Dialogflow for our application is the ability to accurately classify intents, even if the text does not match exactly. For instance, questions such as *“Could you tell me who the author is?”* and *“What’s the name of the author?”* correspond to the same intent, and the system can be trained to extend its database of knowledge by providing additional examples of sentences to recognize. One limitation is that the language has to be known in advance, and this is selected by the users at the start of each tour. In our trials we chose to support only Italian and English, and the intents for both languages were manually added to Dialogflow. It should be noted that, due to the nature of the dialogue system, it would be trivial to add support for additional languages.

Speech synthesizer: the final block of the pipeline is the speech synthesizer, which is also implemented via Google Cloud Services. The speech synthesizer converts the text which is associated with each state included in the Dialogflow agent to an audio stream. The speech synthesizer is also invoked when the robot has to describe an artwork and it is called by the tour manager so that some specific actions (e.g., the movements of the arms to indicate an opera) are synchronized with speech, according to the tour configuration.

## 5 Experimental trials and lessons learned

The application was evaluated during two trial sessions of 1 week of continuous operation each, each preceded by 1 week dedicated to setup and experimentation. The trials took place in two different museums located in Turin, Italy: the Galleria d’Arte Moderna (GAM) and Palazzo Madama. For both locations, the tour guide robot was operating on a single floor, selected by museum personnel, that was mapped beforehand. The robot was operating for the whole week during opening hours, and the visitors who showed interest were invited to take a free tour. Both tours lasted for 30 min on average. A number of safety precautions were taken in order to avoid collisions in case of software or hardware failures. An operator was always equipped with a joystick with a fail-safe button, connected via WiFi directly to the robot, to stop navigation in case of failures. Moreover, a hardware switch to stop all motors was also present on the robot as a last safety measure.

**FIGURE 9:**
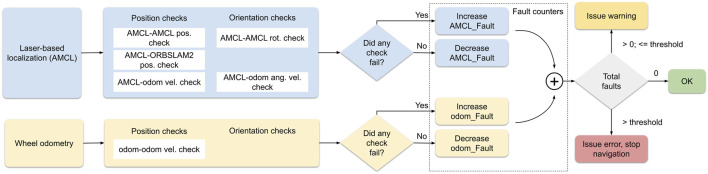
The localization introspection module. The three localization streams are represented with different colors. For each stream, the consistency checks are divided into independent position and orientation checks. Faults from each stream are accumulated on order to make a decision on the localization state.

The first trial was performed in the first floor of GAM. The guided tour consisted of visiting six operas of interest identified in advance by museum personnel. The tour guide robot would guide visitors to each of the selected operas, give a short description, then interact with them by encouraging visitors to ask any of a set of pre-defined questions related to the installation. The robot would then move to the next opera, giving additional information while traveling. At the end of the tour, the robot would thank the visitors and let them know they could continue their visit on their own. The total duration of each tour was ranging from 20 to 40 min, depending on how many questions the visitors asked during the tour, along a path of approximately 150–200 m in length. The second trial was performed in the ceramics gallery, at Palazzo Madama. The structure of the tour was substantially similar to the one in GAM; the only difference was the number of points of interest (eight showcases). For each point of interest, the robot provided a main artwork description, and the visitors were able to ask three additional questions (details about the author, about the historical period, about the execution technique). The duration of a tour in the ceramics galley was approximately between 20 and 45 min (based on questions from the visitors) for a total length of 70 m. The points of interest and the placement of 5G antennas in the two museums is shown in Figure 9.

**FIGURE 10:**
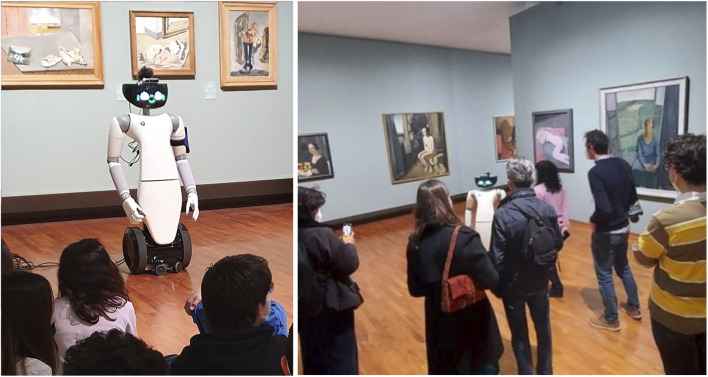
The robot in action during the trials in GAM.

**FIGURE 11:**
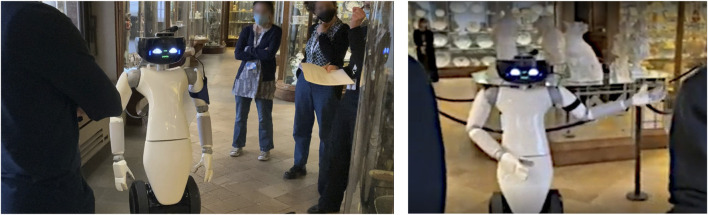
The robot in action during the trials in Palazzo Madama.

The trials were successful: both individual visitors and schools enjoyed following the robot and interacting with it. localization performances were robust for the duration of the trials. In particular, no navigation issues were noted during the week of the trial. Path planning and obstacle avoidance worked reliably, although in two occasions the robot moved too close to obstacles and this was shown to be because of network bandwidth issues. In both cases the robot was stopped by the operator. Examples of the tour guide robot guiding groups of visitors are shown in Figures 10, 11.

### 5.1 Acceptability and engagement with visitors

Visitor satisfaction was evaluated via an anonymous and optional questionnaire at the end of each tour. Feedback was collected from more than 100 people. More details are reported in the Appendix A. Similarly to previous studies (Gasteiger et al., 2021), the tour guide robot was found to be acceptable by the visitors for use within the museum setting, with good visitor engagement and interaction with the robot guide as well as interaction and engagement between the visitors. Also similarly to previous results (Pitsch et al., 2011; Nieuwenhuisen and Behnke, 2013), children in particular were positively engaged by the presence of the robot, and often surrounded it more closely than adult visitors. Engagement tended to be higher at the start of the tour, while some visitors started to explore the installations more and more by themselves as the tour proceeded.

The largest complaint was directed to speech interaction, which sometimes presented delays or not responsive enough (see Section). Similar results were found in another work (Polishuk and Verner, 2017). A recent study (Yousuf et al., 2019) found that visitors feel more engaged if the robot moves and directs its gaze to whoever is speaking, without perceivable delays. However we found that, since our implementation of gaze direction does detect people gazing at the robot’s face but is not able to detect which person is speaking, when many visitors were surrounding the tour guide robot its face orientation tended to switch from person to person.

### 5.2 Ethical and privacy issues

In terms of data collection and privacy, only distance sensors were used for the application (2D LIDAR for localization, mapping and obstacle avoidance; depth camera for obstacle avoidance). For gaze detection, keypoints were extracted from camera images and no RGB images were collected. No sensible data on the users was collected aside from the optional and anonymous questionnaire at the end of the tour.

### 5.3 Hardware and software issues

R1’s onboard battery has a capacity of around 1 h while the robot is moving. This made battery charge a bottleneck for the application, since both tours lasted around half an hour. Occasionally, some visitors or groups of visitors were unable to follow a guided tour because the robot was docked for battery recharging. This issue would be easily solved by employing multiple robots. Robots could in principle run concurrently, although this would require more orchestration at the tour operational level, e.g., for avoiding conflicts such as multiple robots competing for the same opera.

The challenges with speech recognition and voice interaction in the wild are for a large part in separating speech from other noises, and the voice of other people that are not interacting with the robot. One of the problem we encountered in particular, was the noise made by the robot itself during movement. To avoid this, the microphone of the robot was muted during navigation and whenever the arms were moving. This in turn created some difficulties to visitors, because it was not always clear to them when the robot was listening to speech requests. To help visitors to understand when to ask questions or reply to the robot, we programmed the LEDs of the face of the robot to turn green or red to signal when the robot was respectively listening or not. Despite this technical solution, some visitors had difficulties to understand the correct timing when verbally interacting with the robot. Together with background noise and noise due to reverb in the museum large spaces, this was one of the main source of errors during the verbal interaction between visitors and the robot.

The placement and type of microphone used has showed crucial for operation. A microphone with a narrow directional pattern was chosen to mask noises coming from other visitors around the robot. The microphone was pointed in front of the robot’s face, assuming the visitor would face the robot while asking questions or answering. This was combined with a VAD module, that uses a neural model to separate speech from other noises. Consistently, the robot would not understand the answer of a visitor, requiring to repeat or reformulate the answer one or multiple times. This shows how speech recognition in the wild still needs consistent improvements in order to be usable for robot guides interacting with humans.

One open issue in tour guide robot applications is that of maintenance. There was one mechanical failure in one of the arms joints during one trial, requiring a number of guided tours to continue without the ability of the robot to use gestures to point at certain operas.

### 5.4 Network connectivity performance and issues

For the trials, both sites were instrumented with antennas in order to offer 5G coverage of the respective floors where the guided tour takes place. The instrumentation was evaluated in terms of video quality, smoothness of robot navigation, end-to-end delay of audio and video streams. Comparative tests were also carried out using the project’s dedicated 5G connectivity, existing 4G connectivity and, where available, an infrastructural WiFi connection. For the tests, the robot was connected to the 4G and 5G networks through a smartphone connected via USB to the internal router; the robot was controlled by a remote PC connected via cable to the 5G infrastructure. The same route spanning the whole floor was followed by the robot for all experiments. Confirming the results obtained in the preliminary tests, mobile networks showed a marked improvement in both qualitative and quantitative performance compared to WiFi. The *Round-Trip Time* (RTT) of the WiFi connection varied between values of slightly below 100 ms to peaks of 0.5s or more, exhibiting great variance. Bitrate also undergoes large fluctuations. On the other hand, on the mobile network solution designed for this application, the RTT showed to be more stable, with values between 80 ms and 150 ms for the 4G network, and between 10 ms and 100 ms for the 5G network. Additionally, the stability of 4G and 5G connections proved to be more consistent across the whole floor.

The measured maximum throughput in GAM, from a PC onboard the robot to the remote server, was 400 Mbps for downlink and 40 to 65 Mbps for uplink. For comparison, the maximum measured downlink between the 5G smartphone located on R1’s arm and the remote server was 400 Mbps to 1Gbps, indicating the onboard router is limiting the maximum download bandwidth. When performing the same test through the VPN, there was a difference between UDP and TCP connections, with TCP downlink further limited to 300Mbps, and TCP uplink to 35 Mbps. 5G mobile networks (NSA) are asymmetric in terms of available download and upload bandwidth, typically offering much larger download bandwidth compared to upload. This is an issue for a robotic application, which has the opposite requirement of uploading large amounts of sensor data from the robot. In this sense, the network upload bandwidth becomes an upper bound for the amount of transmitted data and must be taken into consideration at the design phase. Due to sensor data compression and throttling that were implemented (more details in Section 4.2.4), during the trials the data transmitted and received by the robot was between 4Mbps and 6Mbps in upload, and between 15Mbps and 20 Mbps in download, falling well between the operational limits of the network infrastructure.

The measured latency towards different machines during the trials is shown in Table 3. The mean latency experienced by the robotic application was 23.5 ms during operation, with a standard deviation of 15 ms. The VPN, after optimization of its parameters, did not affect latency by any appreciable degree.

**TABLE 3:** Latency (in ms) measured from the robot’s onboard PC (r1-base in Figure 3) to different machines during the trials.

Target	5G smartphone	Google services	Remote server	VPN server
min	0.5	14.8	22.7	23.5
max	2.8	195.8	125.8	126.9
mean	0.7	29.8	36.4	39.8
std	0.3	14.4	13.1	14.7

Network congestion was experienced in particular during a time of higher than normal affluence in Palazzo Madama. The coverage implemented in both Museums was design to meet the requirements of the application developed during 5G-Tours Project. As expected, during the peaks of visitors’ affluence to the Museums, the network load increased considerably and the service availability was adapted consequently. From the mobile network perspective this condition would be managed by the Slice mechanism, which was not foreseen for this project designed solution.

Depending on the specific designed coverage and by the signal strength, in few cases the 5G smartphone switched to the 4G service, working in a different network condition: in case of reduced bandwidth availability (about 1Mbps in up-link), the robot stopped the tour. When available again, the automatic attach to the 5G service was driven by the mobile device, and required in some cases operator intervention on the phone. This behavior is expected to be optimized by using an integrated 5G-router.

### 5.5 Navigation performance and issues

The main challenge in performing navigation in the GAM museum was due to it’s size and relative lack of geometric features. During preliminary experiments, we found out that it was not possible to create an accurate map of the floor using the original LIDARs installed on R1, with a usable range of 5 m that was often not enough to see any wall or obstacle around the robot while traveling. This issue is made worse by the necessity to keep a safe distance from operas, thus having the robot move in the center of the corridors. For this reason, a first workaround was to add infrastructure to the floor plan by placing small buckets across the corridors, to augment visible geometric features during SLAM. Later, the onboard LIDARs were upgraded in order to offer a higher usable range. The issue was not, however, impacting the localization accuracy; the AMCL algorithm was able to keep a consistent estimate of the robot’s position with both LIDAR configurations.

**FIGURE 12:**
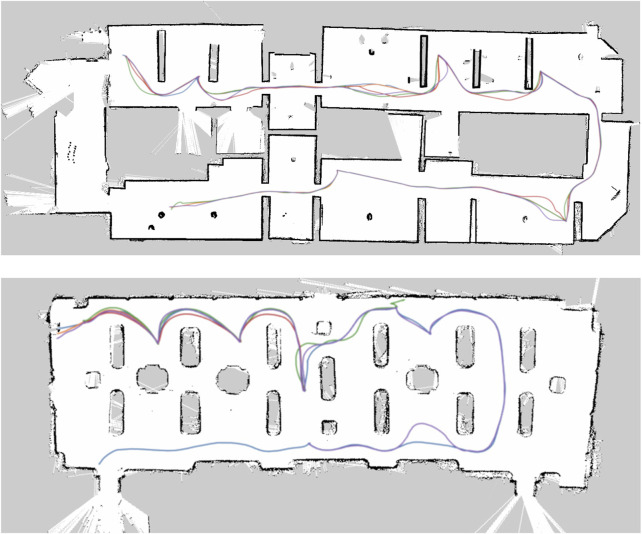
Robot trajectories for a subset of tours in GAM and Palazzo Madama. It is possible to notice a larger variance in the trajectory along the first part of the tour in both GAM and Palazzo Madama, due to more visitors surrounding the robot as well as a larger number of followers on average.

Mapping did not present any issues in Palazzo Madama. A difference from GAM in terms of navigation was the size of the environment. Although the environment could seem more challenging in terms of localization due to the presence of many glass display cases across the room and its walls (Figure 11), their presence was not actually affecting the navigation system, since R1’s LIDARs were placed at a sufficiently low height with respect to the ground to be able to detect the wood basements of the display cases. If this was not the case, a different means of localization would have to be utilized, such as a visual SLAM approach (e.g., ORB-SLAM2) that was used during the preliminary tests. In Table 4, we report the localization performances in terms of Root Mean Square Error (RMSE) at each point of interest between the POI position defined in the tour and the estimated position of the robot at the same POI. This shows how the robot was able to consistently reach each POI across all tours, and be in the correct position to point to operas while explaining. We also show in Figure 12 an example of the tour trajectories over the span of half a day. It can be seen from the image that the trajectories between POIs vary slightly in the first part of the tour (upper side of the map) and this is consistent with the higher engagement by visitors at the beginning of the tour, sometimes standing in the robot’s path. After the last POI, visitors usually departed from the robot, leaving it free to move.

**TABLE 4:** localization performances in terms of RMSE at different POIs of the tour (in m).

POI	1	2	3	4	5	6	7	8
	0.07	0.1	0.13	0.1	0.1	0.15	0.1	0.1

It can be noticed that the sixth POI in Palazzo Madama (12) shows one of the robot trajectories wandering off close to a wall. This was the only navigation failure case in Palazzo Madama, and it was determined to be the effect of the decreased bandwidth availability due to the design of the experimental network: this specific zone was covered by 4G signal only, with an insufficient bandwidth for data transmission, as mentioned later in 5.6. In particular, the navigation component was not able to receive data from the robot in time and to send correct velocity commands to it. In this case, the robot was safely stopped by the operator from the joystick, that is connected directly to the robot and can stop the wheel motors. A further fail-safe measure would have been to push the emergency stop button on the robot’s back.

On the visitors side, another difference between of the two tours was their length. The gallery of ceramics in Palazzo Madama was much smaller in size compared to the large artwork collection of GAM, therefore the travel time from one installation to the next one was shorter, giving the impression of a more dense and detailed tour.

### 5.6 Open challenges with tour execution

The experiment showed the advantages of the 5G network solution performances compared with previous mobile generation (4G). However, the implemented solution for this project is based on the NSA architecture, and did not adopt the Slice management, that would be able to grant constant bandwidth. Mobile Coverage of the area was designed to satisfy the experimental requirements and to explore the challenges of deployment in the field, including the most critical circumstances in which the robot stops to operate.

This challenge could also be addressed from the standpoint of autonomous navigation, via sliding autonomy approaches, shifting from the full navigation setup to a fallback navigation setup running locally on the robot, with limited sensing, able to run on the limited onboard hardware.

## 6 Conclusion

We presented the development choices and findings related to the deployment of an autonomous tour guide robot as part of the 5G-TOURS EU project. The whole application was developed around the concept of exploiting 5G connectivity to off-load part of the computation from the robot itself, testing the feasibility of moving some services that are usually close to the hardware such as obstacle avoidance to a remote machine. This was proved possible due to the low latency of 5G as well as the particular design of the application. The application was deployed in two trials in the field, with a total operational time of 10 days. The experiences collected during the trials showed us that 5G-instrumented environments are an enabler for robotic applications that require streaming of high-bandwidth data such as video for remote processing and for cloud service access. In our application, safe 3D obstacle avoidance and engagement with visitors via gaze detection was possible because of the 5G network, but future tour guide robots that will rely heavily on deep learning techniques that require streaming of raw sensor data for both navigation and natural interaction will benefit from low-latency and relatively constant throughput. The results show how autonomous navigation in a dynamic environment and managing of the tour are possible, and confirm previous findings on how humanoids are a good choice for interacting with visitors, as they can provide natural modes of operation such as voice, gestures, and facial expressions. Some open challenges that were exposed by the trials are speech recognition in the wild, and the need for good network coverage and hardware that can fully exploit 5G technology.

## Figures and Tables

**FIGURE B1:**
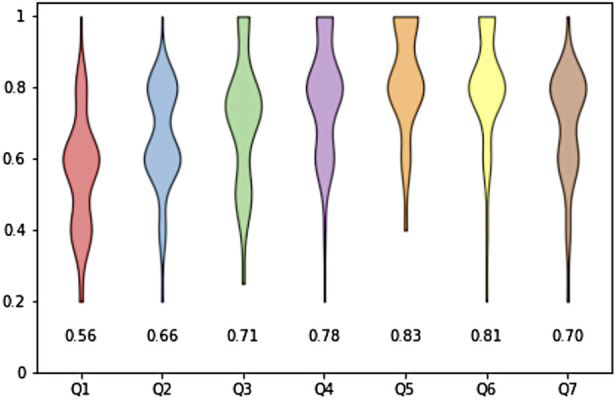
Aggregated QoE results for the final trials in GAM and Palazzo Madama.

## Data Availability

Publicly available datasets were analyzed in this study. This data can be found here: https://github.com/hsp-iit/tour-guide-robot.

## Appendix A:

Questionnaire for visitors

The trials obtained feedback from over 100 people during the days operating in GAM and Palazzo Madama. The questions were formulated based on the *Quality of Experience* (QoE) evaluation methodology, which focuses on the entire service experience. The specific evaluation methodology was developed during the course of the 5 g-TOURS project. The seven questions are shown below:

• Q1: Please state how much you agree with the following statement: the telepresence guide is better than the traditional audio guide.
• Strongly agree, agree, neutral, disagree, Strongly Disagree
• Q2: How pleasant was the user experience in terms of responsiveness of the service?
• Strongly Excellent, Good, Fair, Poor, Very Poor
• Q3: How pleasant was the user experience in terms of intuitiveness of the service?
• Strongly Excellent, Good, Fair, Poor, Very Poor
• Q4: How pleasant was the user experience in terms of usefulness of the service?
• Strongly Excellent, Good, Fair, Poor, Very Poor
• Q5: Please state how much you agree with the following statement: I feel comfortable interacting with and close to the robot during my visit in the museum.
• Strongly agree, agree, neutral, disagree, Strongly Disagree
• Q6: Please state how much you agree with the following statement: I would like to pay an extra fee for the usage of the augmented tourism experience.
• Strongly agree, agree, neutral, disagree, Strongly Disagree
• Q7: Please state how much you agree with the following statement: Your interaction with the museum contents has been stimulated and you felt deeply involved in the artistic context.
• Strongly agree, agree, neutral, disagree, Strongly Disagree
• If you like, please provide your open feedback on your experience during the Museum visit:
• “Open comment”

The questionnaire results are shown in Figure B1.
